# Data on preparation of psychrotolerant bacterium *Shewanella olleyana* sp. nov. cells for transmission electron microscopy

**DOI:** 10.1016/j.dib.2016.09.049

**Published:** 2016-10-06

**Authors:** Chitho P. Feliciano, Windell L. Rivera

**Affiliations:** a Microbiological Research Laboratory, Biomedical Research Section, Atomic Research Division, Philippine Nuclear Research Institute, Department of Science and Technology (PNRI-DOST), Diliman, Quezon City, Philippines; b Institute of Biology, College of Science, University of the Philippines, Diliman, Quezon City, Philippines; c Natural Sciences Research Institute, University of the Philippines, Diliman, Quezon City, Philippines

**Keywords:** Transmission electron microscopy, *Shewanella olleyana*, Ultrastructure, Psychrotolerant bacterium

## Abstract

This data article contains transmission electron microscopy (TEM) images of psychrotolerant bacterium *Shewanella olleyana* sp. nov. Cells of *S. olleyana* were grown following an optimized culture conditions in liquid medium. Procedure for the preparation of cells suitable for TEM is described in detail.

**Specifications Table**

**Table:** 

Subject area	Microbiology
More specific subject area	Microscopy
Type of data	Transmission electron microscopy (TEM) images, Tables
How data was acquired	TEM following an optimized cell preparation protocol
Data format	Analyzed
Experimental factors	Bacterial cells were grown at different temperatures in liquid and solid media
Experimental features	Culture conditions and preparation for electron microscopy of the bacterial cells were optimized
Data source location	N/A
Data accessibility	Data are provided in this article

**Value of the data**

• These data describe the culture conditions and preparation of *Shewanella olleyana* cells suitable for TEM.
• The obtained TEM images of *S. olleyana* can serve as reference data for its morphological features and ultrastructural characteristics.
• To our knowledge, this data article is first to report the ultrastructure of *S. olleyana.*
• The data presented here confirmed the presence of high levels of iron and/or iron sulfide in the broth culture precipitates of *S. olleyana*. Researchers using this psychrotolerant bacterium can take these findings into consideration for its culture propagation and other purposes (e.g. microscopy).

## 1 Data

The data presented in this article show the morphological and ultrastructural characteristics of psychrotolerant bacterium *Shewanella olleyana* sp. nov. obtained by TEM. Cells were grown and prepared following an optimized procedure. *S. olleyana* cells were grown and propagated in solid medium at temperatures between 4 and 10 °C for 24–48 h. Complete sedimentation of suspended *S. olleyana* cells (grown in solid medium) was achieved following centrifugation at 4000 x *g* for 10 min at 4 °C. The presence of residual or contaminating reduced form of iron and/or iron sulphide was detected when cells are grown in liquid medium.

## 2 Experimental design, materials and methods

### 2.1 Bacterial strain, culture conditions, and cell preparation

*Shewanella olleyana* sp. nov. (LMG 21437) was obtained from BCCM/LMG Bacteria Collection, Laboratory for Microbiology, University Ghent K. L. Ledeganckstraat, Belgium. *S. olleyana* strain was grown routinely in Zobell׳s marine agar 2216 [1]. To determine the optimum growth conditions for the culture and propagation of *S. olleyana,* its growth response to varying incubation temperatures (4–10, 25, 30, and 35 °C) was observed for 24–48 h (Table 1, Table 2).

**Fig. 1:**
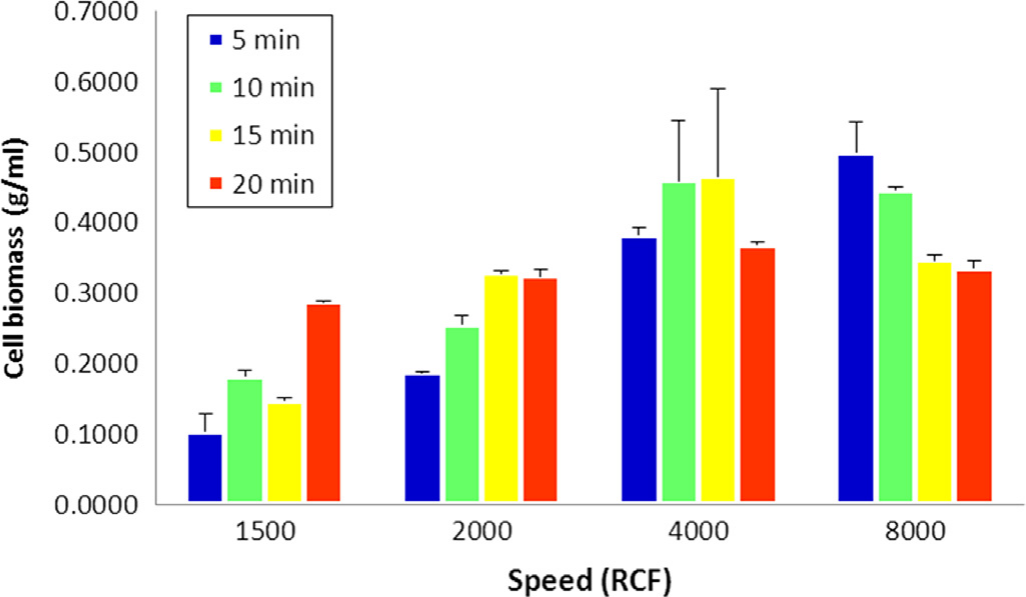
Graphical presentation of the results obtained from centrifugation studies. Data are presented as average yield of cell biomass in gram per ml of culture broth.

The minimum centrifugation speed and time required to sediment *S. olleyana* cells suspended in MH buffer ((280 mM mannitol/10 mM HEPES (4-(2-hydroxyethyl) piperazine-1-ethanesulfonic acid) pH 7.4) at 1:1 ratio (w/v) was determined. Immediately after harvesting the cells from the agar plates and suspending it in MH buffer, the cell suspension was subjected to varying centrifugation speed (1500, 2000, 4000, and 8000 x *g*) and sedimentation time (5, 10, 15 and 20 min), as shown in Fig. 1.

For TEM analysis, 24–48 h old *S. olleyana* cells were harvested from agar plates. Cells were flooded with 4 ml of sterile cold physiological saline (0.85% NaCl) and scraped using a glass rod to detach the cells. The cell suspension was centrifuged at 4000 g for 10 min at 4 °C. The resulting pellet was washed with cold physiological saline. Cells were resuspended in MH buffer and stored at −86°C until use. Growth of *S. olleyana* cells in marine broth medium was also observed.

### 2.2 X-ray fluorescence (XRF) spectroscopy analysis of *S. olleyana* precipitates from broth cultures

**Fig. 2:**
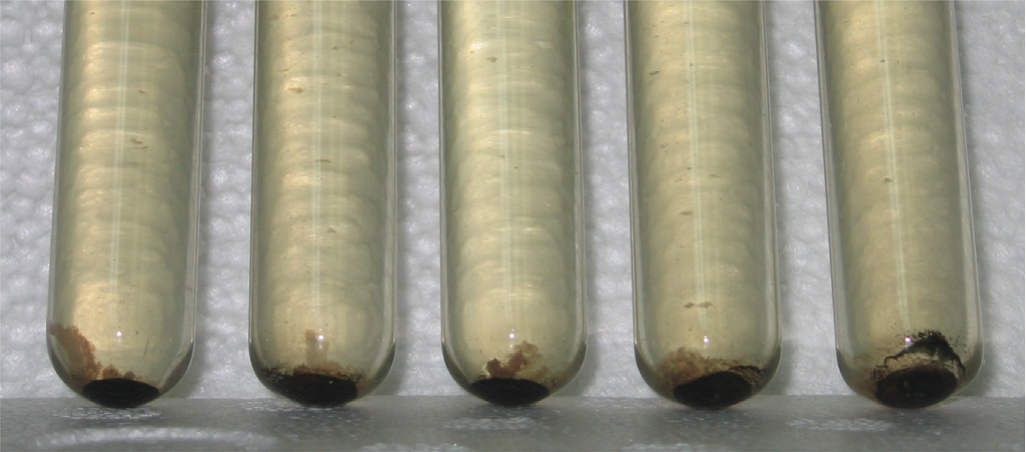
*S. olleyana* grown at 4–10 °C on Zobell׳s marine broth. Black precipitates formed at the bottom of the culture tubes after 48 h incubation.

**Fig. 3:**
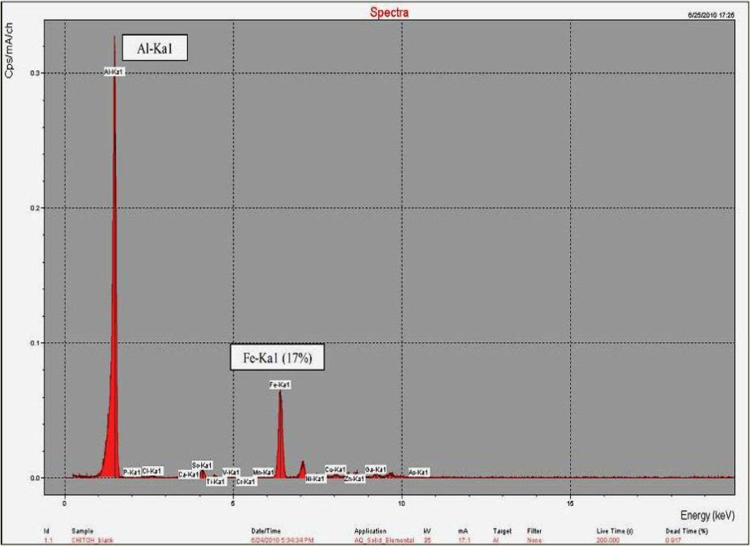
XRF spectrum of black precipitates obtained from the broth cultures of *S. olleyana*. Al-Ka1 = background spectra of aluminum dish where the precipitate was adhered for analysis.

To verify the occurrence of iron reduction in the broth medium by *S. olleyana* cells, XRF analysis of the recovered precipitates was done. The PANalytical Epsilon5 EDXRF spectrometer was used for the multi-elemental analysis of the sample. The instrument is equipped with 600 W-anode x-ray tube and 100 kV generator, up to 15 secondary targets and a high resolution PAN-32 detector. To identify the elemental constituents of the sample, it was analyzed qualitatively using the spectrum generated by the EDXRF. Cells were grown on 10 ml marine broth [1] incubated at 4–10 °C for 3–5 days. Formation of black precipitates during incubation was observed (Fig. 2). Precipitates were recovered and dried free of moisture at 110 °C for 14 min in an aluminum dish. The precipitate that adhered on the aluminum dish was placed on top of an “XRF insert”, covered with X-ray thin film sample support and inserted in stainless steel cap. The sample assembly was then put inside the EDXRF spectrometer and analyzed using the PANalytical Epsilon Software for elemental analysis. Fig. 3 shows the result of XRF analysis of the black precipitates. The precipitates contain high levels of elemental iron (17%), confirming that this could be a mixture of reduced form of iron (Fe^2+^) and/or iron sulphide precipitates produced by *S. olleyana.*

### 2.3 TEM and negative staining of *S. olleyana*

**Fig. 4:**
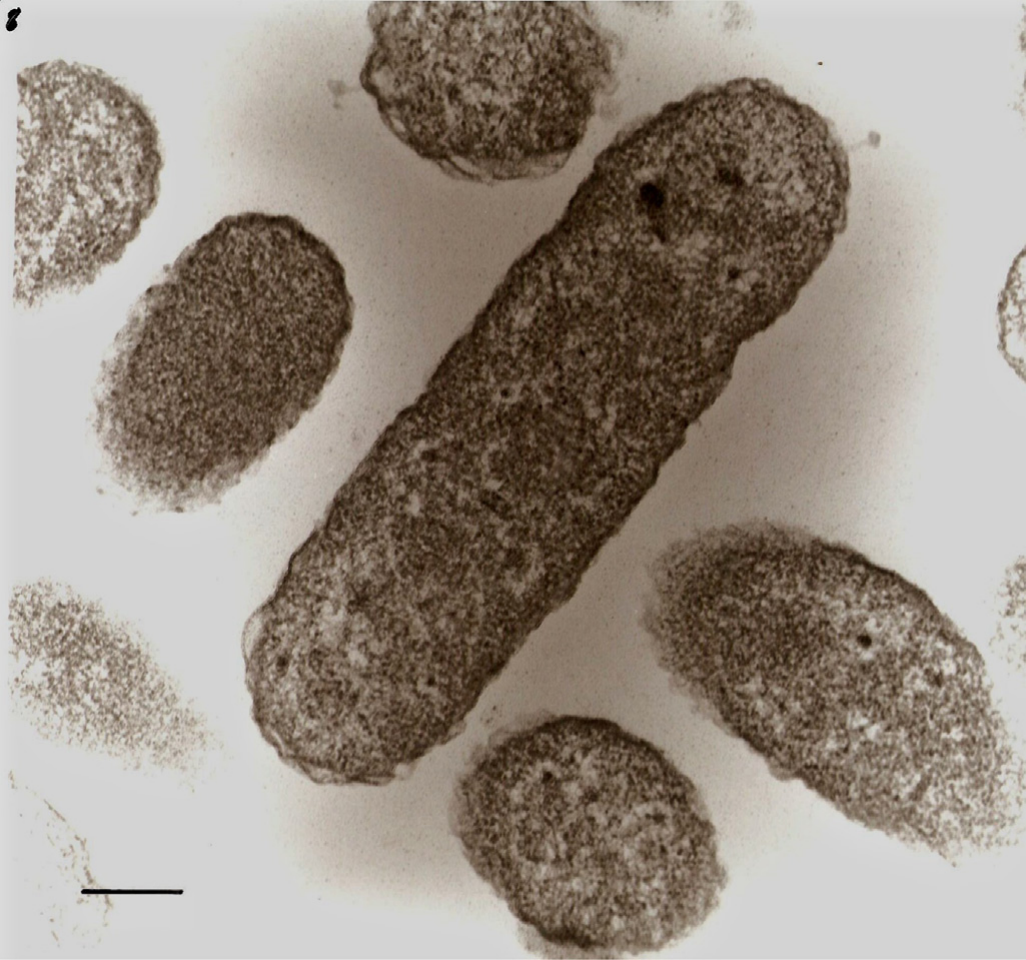
Transmission electron micrograph of intact *S. olleyana* suspended in MH buffer and stored at −86°C. Bar, 0.25 μm.

**Fig. 5:**
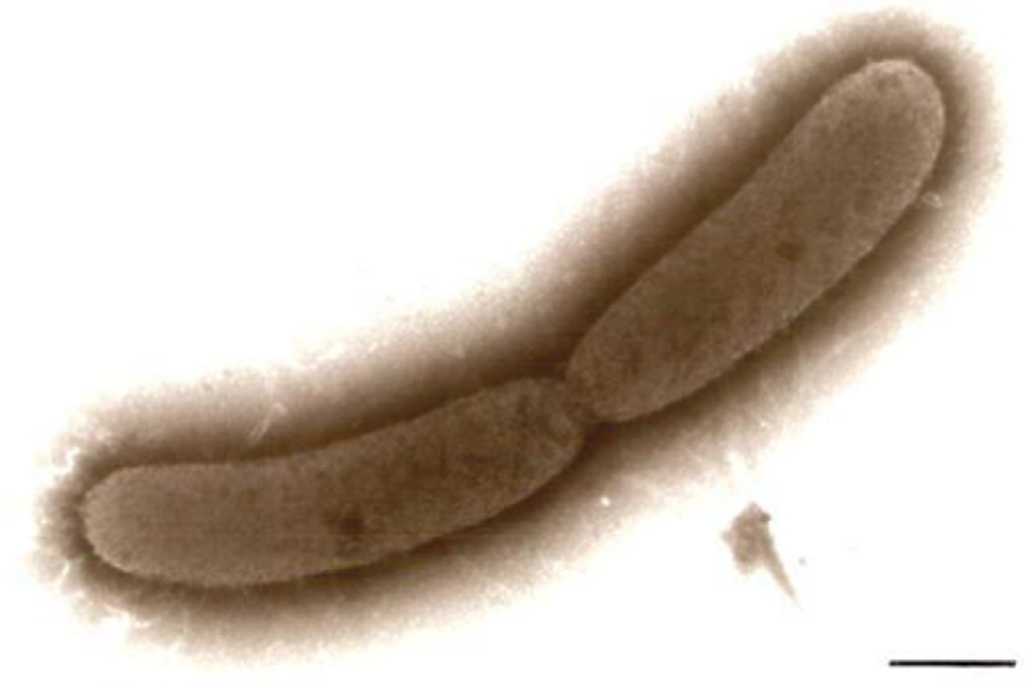
Transmission electron micrograph of negatively-stained *S. olleyana* undergoing cell division (Bar, 0.5 μm).

**Fig. 6:**
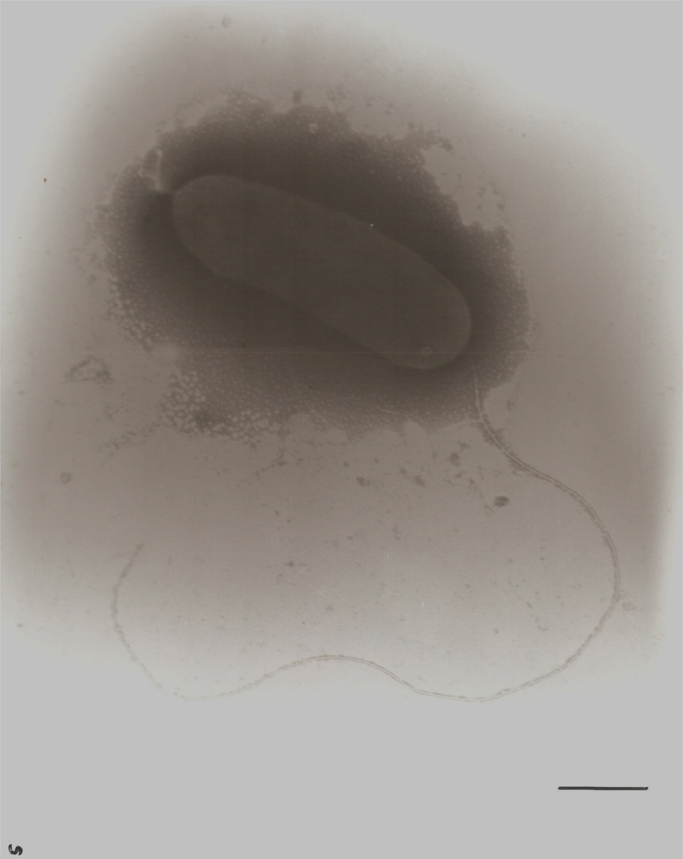
Transmission electron micrograph of negatively-stained *S. olleyana* showing a single polar flagellum (F). Bar, 0.5 μm.

*S. olleyana* cells were fixed in buffered 2.5% glutaraldehyde and 4% paraformaldehyde. Cells were washed three times with physiological saline to remove excess fixative and were fixed in unbuffered 1% osmium tetroxide and washed with physiological saline. It was then dehydrated in a graded series of acetone solutions and gradually impregnated in Epon resin with heat polymerization. Semi-thin survey sections were sliced with glass knives, stained with toluidine blue and used to orient sections. Ultra-thin sections were mounted on uncoated copper grids and stained with uranyl acetate and lead citrate. Sections were examined and viewed in a JEOL 1010 TEM. Negative staining of *S. olleyana* cells was done following a previously published method [2]. Samples were viewed using a JEOL 1200 EX electron microscope (Fig. 4, Fig. 5, Fig. 6).

### 2.4 Statistical analysis

Each data point represents the mean+SD of two trials. GraphPad InStat software was used to determine the differences among the means. Data were compared using one-way analysis of variance (ANOVA) with post-test. Dunnett׳s test was used to compare treatment means against the control mean. Statistical significance was determined at *p*<0.05.

## Figures and Tables

**Table 1:** Growth of *S. olleyana* in Zobell׳s marine agar medium incubated at different temperatures. Growth was grouped into A+=excellent growth (>300 cfu/plate); A=good growth (250–300 cfu/plate); B = fair growth (1–100 cfu/plate); and NG = no growth (0 CFU/plate).

Test temperatures (°C)	Growth of *S. olleyana*	
	After 24 h	After 48 h
4–10	A	A+
25	B	B
30	NG	NG
35	NG	NG

**Table 2:** Yield of cell biomass per culture plate after 48 h incubation at 4–10 °C.

No. of trials	No. of plates	Total yield (g)	Yield per plate (g)
I	14	3.24	0.231
II	11	2.13	0.194
III	13	1.48	0.114
IV	6	1.42	0.237

## References

[1] Zobell C.E. (1941). Studies on marine bacteria. I. The cultural requirements of heterotrophic aerobes. J. Mar. Res..

[2] Allan V.J.M., Callow M.E., Macaskie L.E., Paterson-Beedle M. (2002). Effect of nutrient limitation on biofilm formation and phosphatase activity of a *Citrobacter* sp. Microbiology.

