# Supplementary material for: Data on preparation of psychrotolerant bacterium Shewanella olleyana sp. nov. cells for transmission electron microscopy
Source: Data Brief. 2016 Oct 6;9:710–5. doi: 10.1016/j.dib.2016.09.049 (PMC5094100; doi:10.1016/j.dib.2016.09.049)
Supplement: Supplementary file 1 — Supplementary material [file mmc1.docx]

**Conflict of Interest**

We have no conflict of interest to declare.

**SIGNED**

Chitho P. Feliciano

First and Corresponding Author
